# Incorporating methylation genome information improves prediction accuracy for drug treatment responses

**DOI:** 10.1186/s12863-018-0644-5

**Published:** 2018-09-17

**Authors:** Xiaoxuan Xia, Haoyi Weng, Ruoting Men, Rui Sun, Benny Chung Ying Zee, Ka Chun Chong, Maggie Haitian Wang

**Affiliations:** 1 Division of Biostatistics, Centre for Clinical Research and Biostatistics, JC School of Public Health and Primary Care, The Chinese University of Hong Kong, Shatin, N.T, Hong Kong, SAR China; 2 CUHK Shenzhen Research Institute, Shenzhen, China

**Keywords:** Methylation, Prediction, SNPs, Neural network, Treatment responses

## Abstract

**Background:**

An accumulation of evidence has revealed the important role of epigenetic factors in explaining the etiopathogenesis of human diseases. Several empirical studies have successfully incorporated methylation data into models for disease prediction. However, it is still a challenge to integrate different types of omics data into prediction models, and the contribution of methylation information to prediction remains to be fully clarified.

**Results:**

A stratified drug-response prediction model was built based on an artificial neural network to predict the change in the circulating triglyceride level after fenofibrate intervention. Associated single-nucleotide polymorphisms (SNPs), methylation of selected cytosine-phosphate-guanine (CpG) sites, age, sex, and smoking status, were included as predictors. The model with selected SNPs achieved a mean 5-fold cross-validation prediction error rate of 43.65%. After adding methylation information into the model, the error rate dropped to 41.92%. The combination of significant SNPs, CpG sites, age, sex, and smoking status, achieved the lowest prediction error rate of 41.54%.

**Conclusions:**

Compared to using SNP data only, adding methylation data in prediction models slightly improved the error rate; further prediction error reduction is achieved by a combination of genome, methylation genome, and environmental factors.

## Background

Increasing evidence reveals the important role of epigenetic factors in explaining the etiopathogenesis of human diseases, especially in cancer [1]. For example, Chaudhry et al. verified that *BRCA1* promoter methylation was useful in predicting the response to chemotherapy in epithelial ovarian cancer [2], and Shindo et al. found that a high methylation M-score was a significant risk factor for recurrent bladder cancer [3]. Diseases other than cancer have shown profound alterations in DNA methylation profiles [4, 5]. Consideration of the effect of epigenetic factors on disease traits has the potential to improve disease prediction, which has been adopted in several recent empirical studies [6–9]. However, it is still challenging to integrate different types of omics data into prediction models. In addition, there has been insufficient information to precisely clarify the contribution of methylation information to prediction.

In this study, a stratified drug-response prediction model is built based on an artificial neural network (ANN) to identify the contribution of methylation information to predicting the change in the circulating triglyceride (TG) level after fenofibrate intervention. Omics data, including genetic, epigenetic, and clinical factors, are used as predictors. The analysis of GAW20 real data demonstrates that the inclusion of the methylation data improves the prediction accuracy marginally, which provides an indication for future prediction research.

## Methods

### GAW20 data

GAW20 real data were used in this study and were provided by the Genetics of Lipid Lowering Drugs and Diet Network (GOLDN) study, which aimed to identify the genetic determinants of the responses of circulating lipid levels to fenofibrate treatment interventions. In total, 1053 individuals from families with at least 2 siblings were recruited. They all self-reported as being of white ethnicity [10]. TG levels were measured at visits 1, 2, 3, and 4, among which data from visits 1 and 2 were collected before fenofibrate intervention, whereas the other two TG measurements were made after the intervention (visits 3 and 4). At visit 1, participants were measured using a lipid profile after an overnight fast. A repeated lipid file occurred the next day during visit 2. The treatment period lasted 3 weeks, after which participants returned to the clinic for 2 consecutive days for visits 3 and 4 [10]. Meanwhile, DNA methylation levels were measured at visits 2 and 4. DNA was isolated from CD4^+^ T cells harvested from stored buffy coats and the proportion of sample methylation was quantified at > 450,000 cytosine-phosphate-guanine (CpG) sites [10].

### Data quality control

In the quality control process, 39 participant outliers were removed, and only subjects without any missing data for the key variables (TG levels at visits 1 to 4, methylation value at visit 2, and genotypes) were used. A total of 523 participants were included in the analysis. For the genotype data, single-nucleotide polymorphisms (SNPs) with a minor allele frequency < 0.01 were excluded. Missing variants were imputed according to the probability distribution of the genotype in all subjects. For the methylation data, cross-reactive probes and probes containing common variants were filtered. Beta-mixture quantile normalization was used to correct for the Infinium Type I/II bias [11], and participant outliers were identified by hierarchical clustering and Eigenstrat [12].

### Drug-response definition

Drug response was used as the dependent variable which could be defined as the percentage change in the TG level.

$$ TG\kern0.5em change\kern0.5em percentage=\left( TG\kern0.5em post- TG\kern0.5em pre\right)/\left( TG\kern0.5em pre\right) $$

**Fig. 1:**
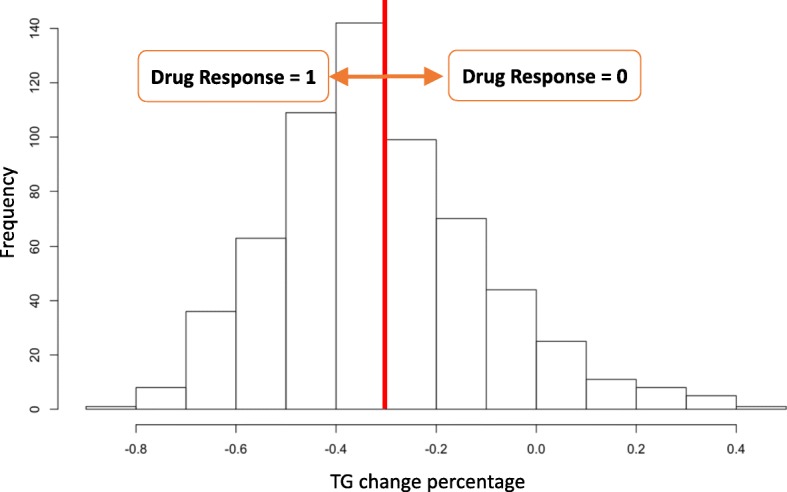
Distribution of percentage change in circulating triglyceride (TG)

Where *TG pre* is the average of TG levels at visits 1 and 2, and *TG post* is the average of TG levels at visits 3 and 4. It was reported that fenofibrate, which was the intervention drug for the GAW20 real data, usually reduced the plasma TG level by approximately 30 to 60% in hyperlipoproteinemia patients at a dosage of 200–400 mg daily [13]. In this regard, we defined the drug-response variable as 1 when the TG level was reduced by more than 30% after treatment, which meant the drug worked for patients. Otherwise, the drug-response variable was coded as 0, which meant that the drug did not work as expected. Consequently, as shown in Fig. 1, 301 and 222 participants were coded as 1 and 0, respectively.

### Stratified variable selection and prediction modeling

The features related to drug response were selected in a stratified manner [14], first within each data type, and then aggregated in an ANN to predict the drug response [15]. ANNs are designed to perform learning tasks using a collection of computational units and a system of interlinking connections [16]. The central idea of ANN is to extract features by linearly combining the inputs and then use nonlinear functions to model the targets. Therefore, a neural network can be thought of as a nonlinear generalization of linear models, which generalizations can be used for classification and regression [17]. We used the AMORE package in the R 3.3.2 GUI 1.68 Mavericks build (7288) to conduct the ANN analysis [15]. The stratification enables precise variable selection within each data type, and the ANN enables the consideration of interaction effects within and across data types [18]. Five-group cross-validation error rates and their standard deviation were calculated to evaluate prediction performance.

The generalized estimation equation (GEE) model was used to select significant SNPs and adjust for family relatedness [19]. CpG sites were selected by linear mixed model (LMM) with an empirical kinship matrix to adjust for family structure [20]. Both the mixed-effect model and GEE are theoretically suitable for the selection of the SNPs and CpG sites while controlling for family structures. The two methods differ in the way they estimate the coefficients and treat the population correlation structure. The major consideration for us was the ability of software packages to handle a binary phenotype, control family structure, and treat continuous random-effect variables. An arbitrary *p* value threshold of 10^− 4^ was applied to filter the biomarkers for GEE and LMM so that a moderate number of predictors can be used in the prediction model. SNPs were pruned to avoid the strong influence of SNP clusters, by snpgdsLDpruning, and the linkage disequilibrium threshold was set at 0.2 [21, 22]. The empirical kinship matrix was calculated using the pruned SNPs to control for family relatedness. Other clinical variables, including sex, age, and smoking status, were also used as predictors.

Predictors were added into the prediction model step-by-step by data types. Afterward, chosen SNPs were inputted into the ANN first, followed by significant CpG sites. Finally, age, sex, and smoking status were included. This stratified method made it easy to identify the respective contribution of each category of information to prediction.

A three-layer ANN was applied with one hidden layer. The hyperbolic tangent sigmoid transfer function was used as the activation function (*a*) for the hidden layer, which has the following form:

$$ a= transig(n)=-1+2/\left(1+{e}^{-2n}\right) $$

A linear function was used as the activation function for the output layer (*purelin*):

$$ a= purelin(n)=n $$

The learning rate and global momentum were set at 0.01 and 0.4, respectively. The preferred training method was an adaptive gradient descent with momentum. The least mean squares criterion was used to measure the proximity of the neural network prediction to its target when training the ANN.

## Results

### Contribution of each variable to prediction

**Fig. 2:**
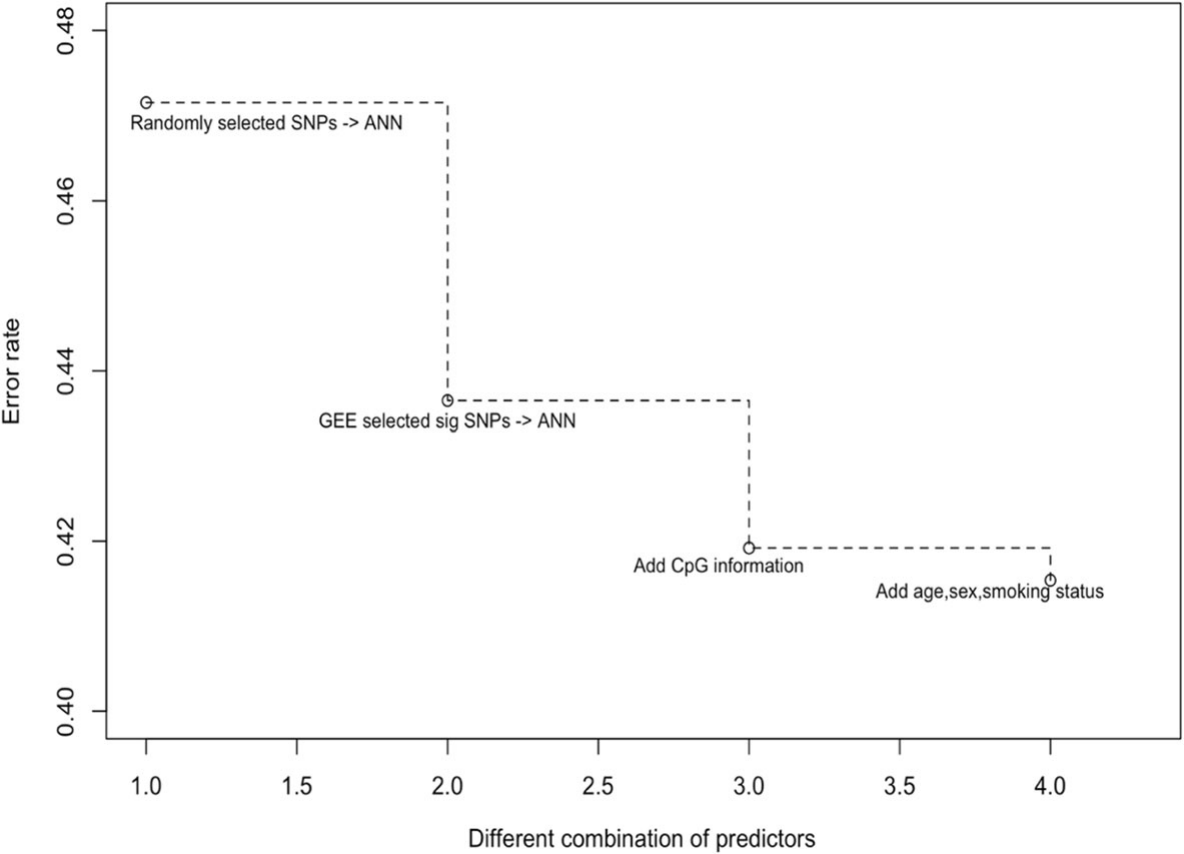
Stratified drug-response prediction model: the error rate improved when adding additional variables

Three types of data (SNPs, methylation, and clinical information) were included in the ANN model in a stepwise manner to compare their contributions to the prediction ability of the model. The baseline model simulates the null scenario; that is, 100 SNPs were selected from the autosomes at random and used to predict the phenotype with the ANN in 5-group cross-validation. This gave a baseline error rate of 47.15% (SD: 3.79%), representing a random-guess prediction error under the ANN. Next, including the SNP information yielded a mean test prediction error rate of 43.65% (SD: 4.79%). When methylation information was added, the prediction model achieved an error rate of 41.92% (SD: 4.64%; Wilcoxon rank sum test *p* value: 0.3759), which implies that the inclusion of methylation information improves the prediction model. When clinical factors (age, sex, smoking status) were also included, the error rate dropped slightly to 41.54% (SD: 5.66%, Wilcoxon rank sum test *p* value: 0.5) (Table 1). Figure 2 shows the changes of prediction error rate using different variable sets. Sequentially adding SNPs, CpG sites, and environmental factors gradually pushed down the prediction error rate.

**Table 1:** Stratified drug-response prediction model incorporating omics data

	Training error rate ± SD	Test error rate ± SD
SNP	8.59% ± 0.88%	43.65% ± 4.79%
CpG	8.88% ± 2.87%	45.00% ± 3.29%
Add useful CpG information to SNPs	0.00% ± 0.00%	41.92% ± 4.64%
Add useful CpG information to SNPs + age, sex, smoking	0.00% ± 0.00%	41.54% ± 5.66%

The error rates are average 5-fold cross-validation error rates by ANN using inputs

### Biological function of identified variables

Finally, we report the biological meaning of variables identified using all data. Many of the identified SNP and CpG markers had functions that are related to the regulation of the circulating level of TG, which is a major storage molecule for metabolic energy [23]. To list a few genes (Tables 2 and 3), *FTO* (rs10521308, *p* value = 9.47E-05) and *CTNNBL1* (rs2206135, *p* value = 7.75E-05) have both been strongly associated with obesity risk and related traits [24, 25]. The gene *DGAT1* (cg13438334, *p* value = 8.49E-05) plays a role in catalyzing the committed step in the biosynthesis of TGs [23], and *ALDH4A1* (cg22390041, *p* value = 4.97E-05) is known to catalyze ester hydrolysis, suggesting that it may lead to a change in the TG level [26].

**Table 2:** Selected SNPs that pass the threshold of 10^− 4^ in the GEE model

SNP	Chromosome	Gene	Position	*p* Value	MAF
rs10521308	16	*FTO*	80,459,640	9.47E-05	0.05
rs2206135	20	*CTNNBL1*	35,914,069	7.75E-05	0.42
rs710711	12	*BEST3*	124,093,552	9.98E-05	0.38
rs7096710	10	*C10orf59*	63,063,177	2.92E-05	0.02
rs4851313	2	*CHST10*	100,395,434	5.47E-05	0.44

*MAF* minor allele frequency

**Table 3:** Selected CpG sites that pass the threshold of 10^−4^ in the LMM model

CpG sites	Chromosome	Gene	Position	*p* Value
cg13438334	8	*DGAT1*	145,550,989	8.49E-05
cg11666857	5	*SLC6A19*	1,207,464	2.44E-05
cg22390041	1	*ALDH4A1*	3,036,916	4.97E-05
cg15883716	1	*ANKRD45*	19,226,319	2.06E-06
cg01056590	1	*CABC1*	173,638,701	4.07E-06

## Discussion

Epigenetic factors are thought to be significantly associated with human diseases, making it plausible to incorporate methylation information for better disease prediction. In this study, we used an ANN to build a stratified drug-response prediction model in which SNPs, methylation, age, sex, and smoking status were considered as predictors. The GAW20 real-data analysis shows that the incorporation of methylation information could reduce the prediction error rate by approximately 4% (*p* value = 0.3759). The combination of significant SNPs, CpG sites, age, sex, and smoking status achieved the best prediction error rate of 41.54%.

In previous studies, Deng et al. used fusing networks to predict schizophrenia from SNPs, methylation, and functional magnetic resonance imaging data [27]. They achieved a 2.8% increase in prediction accuracy, increasing from 52.9% (using SNPs only) to 55.7% (using SNPs and methylation information). We achieved similar improvement when adding methylation information to SNP. Several reasons may account for the difference between our work and theirs. First, the cell type from which they collected methylation information for prediction is different from the GAW20 data. Methylation varies across cell types, and changes in some cell types are more environment and phenotype specific than in other cell types [4]. The GAW20 real data set methylation information was collected from CD4^+^ T cells harvested from stored buffy coats, and the phenotype was the TG level in blood, which has a strong correlation with T-cell functions [10]. Second, family relatedness in the GAW20 real data set played a role in the lower prediction error rate. Third, 208 participants (96 cases and 112 health controls) were recruited in the study by Deng et al., whereas our study has a larger sample size of 523 participants. Finally, the method we applied uses a stratified feature selection and prediction approach. The stratification enables better power to selected variables within each stratum, compared to an all-mixture type of prediction modelling, resulting in an enhanced final prediction accuracy.

## Conclusions

Adding methylation data slightly improved the prediction accuracy for drug response using a neural network based prediction algorithm with GWAS data. The result could be constraint by the source of tissue, the outcome variable and the disorder under study. Further studies in other cohorts are necessary to validate the results.

## Abbreviations

ANN: Artificial neural network
CpG: Cytosine-phosphate-guanine
GAW: Genetic Analysis Workshop
GEE: Generalized estimation equation
GOLDN: Genetics of Lipid Lowering Drugs and Diet Network
LMM: Linear mixed model
SD: Standard deviation
SNPs: Single-nucleotide polymorphisms
*TG post*: The average of triglyceride levels at visits 3 and 4
*TG pre*: The average of triglyceride levels at visits 1 and 2
TG: Triglyceride
